# Coordination of Cell Differentiation and Migration in Mathematical Models of Caudal Embryonic Axis Extension

**DOI:** 10.1371/journal.pone.0022700

**Published:** 2011-07-28

**Authors:** Nigel C. Harrison, Ruth Diez del Corral, Bakhtier Vasiev

**Affiliations:** 1 Department of Mathematical Sciences, University of Liverpool, Liverpool, United Kingdom; 2 Instituto Cajal, Consejo Superior de Investigaciones Científicas, Madrid, Spain; Institute of Science and Technology Austria, Austria

## Abstract

Vertebrate embryos display a predominant head-to-tail body axis whose formation is associated with the progressive development of post-cranial structures from a pool of caudal undifferentiated cells. This involves the maintenance of active FGF signaling in this caudal region as a consequence of the restricted production of the secreted factor FGF8. *FGF8* is transcribed specifically in the caudal precursor region and is down-regulated as cells differentiate and the embryo extends caudally. We are interested in understanding the progressive down-regulation of *FGF8* and its coordination with the caudal movement of cells which is also known to be FGF-signaling dependent. Our study is performed using mathematical modeling and computer simulations. We use an individual-based hybrid model as well as a caricature continuous model for the simulation of experimental observations (ours and those known from the literature) in order to examine possible mechanisms that drive differentiation and cell movement during the axis elongation. Using these models we have identified a possible gene regulatory network involving self-repression of a caudal morphogen coupled to directional domain movement that may account for progressive down-regulation of *FGF8* and conservation of the *FGF8* domain of expression. Furthermore, we have shown that chemotaxis driven by molecules, such as FGF8 secreted in the stem zone, could underlie the migration of the caudal precursor zone and, therefore, embryonic axis extension. These mechanisms may also be at play in other developmental processes displaying a similar mode of axis extension coupled to cell differentiation.

## Introduction

During embryonic development, generation of cell diversity needs to be coordinated with tissue growth in order to achieve the right size, cell number and shape of the different organs. Depending on the developmental context this is implemented differently. Several developmental systems with predominant growth along one axis share a similar strategy: cells at one end of the domain remain undifferentiated and give rise progressively in time and space to cells that have a more restricted fate and can differentiate further. This occurs for example during growth of plant root meristemes, caudal extension of short germ band insects and worms, extension of the vertebrate limb bud, growth of bones, and caudal extension of the vertebrate body axis [1], [2], [3], [4], [5], [6]. In this paper we focus on the latter process, namely we are interested in understanding how the migration and differentiation of cells associated with the caudal extension are controlled at the molecular and cellular level.

Vertebrate embryos display very important differences along their rostro-caudal (head-to-tail) axis from very early stages of development which are manifested, for example, by the orientation and movement of the primitive streak along the rostro-caudal axis. This is a transient structure, composed of cells that form a groove in the epiblast, through which cells ingress to form the mesoderm and the endoderm. The primitive streak displays a rostral tip (named Hensen's node), which has an important pattern organizing role on the cells that develop in its vicinity and influences the primitive streak dynamics. Primitive streak development goes through an initial phase of rostral elongation followed by caudal regression.

**Figure 1:**
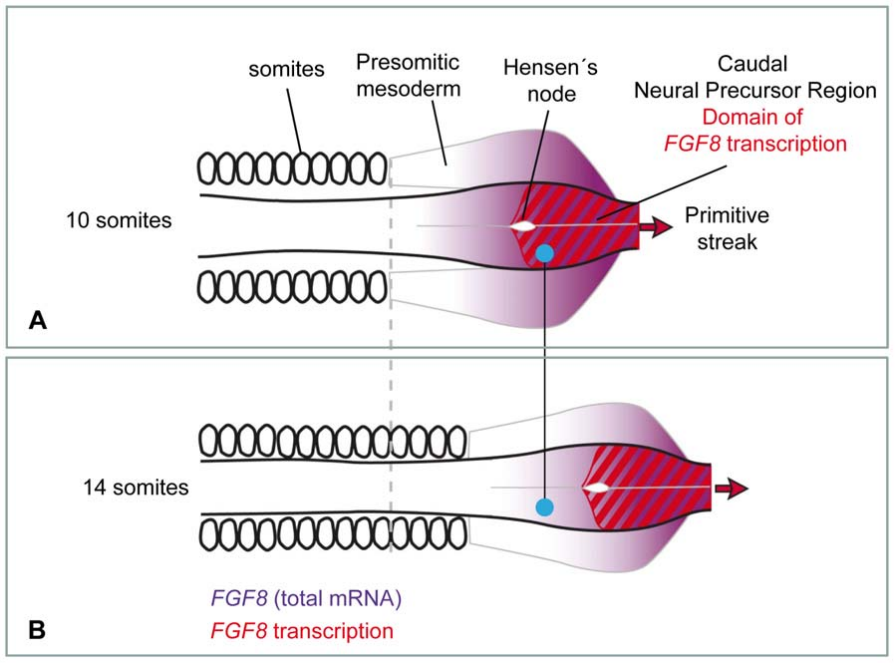
Progressive down-regulation of *FGF8* at the caudal precursor zone. Schematic drawing showing expression of *FGF8* (purple) in embryos of 10 (**A**) and 14 (**B**) somites respectively. Transcription of *FGF8* (red) only occurs at the primitive streak and adjacent epiblast but *FGF8* mRNA extends into the presomitic mesoderm and adjacent neural tube due to maintenance of the transcript as the embryo extends. Cells which are left behind the moving caudal neural precursor zone (blue dot) do not regress caudally and stop transcribing *FGF8*.

Formation and rostral elongation of the primitive streak is associated with cell movements that may have a lateral intercalation component [7] or be of chemotactic nature [8], [9]. Regression of the primitive streak is associated with the movement of a group of cells surrounding and including Hensen's node, that behaves as a precursor region for postcranial mesoderm and neural tube. Although some stem-like cells giving rise to several lineages may reside in this caudal precursor region, different populations have been discovered to give rise preferentially to distinct lineages. The mesodermal layer of Hensen's node gives rise to the notocord while the rostral primitive streak gives rise to somites. The ectodermal layer of Hensen's node gives rise to the floorplate of the neural tube while the ectoderm adjacent to the primitive streak gives rise mainly to lateral (non-floorplate) neural tube [10] and some somitic tissue [11], [12], [13], [14]. Cells in this region proliferate and their daughter cells can either continue to move caudally and remain in the caudal precursor region as the streak regresses or can be left behind and consequently exit this region (Figure 1).

In general, it is thought that cells either remain in the caudal precursor region or transit to a more differentiated state depending on the degree of activation of signaling pathways which in turn depends on their exposure to specific morphogens produced by particular cell populations. A precise molecular marker for this precursor population has not been described, but in the epiblast layer, according to fate maps, it may correspond to cells that transcribe *FGF8* as detected with the *FGF8* intronic probe [15]. We will refer to this population as the caudal neural precursor region (CNPR) (which includes the caudal lateral epiblast [16], and the epiblast layer of the node and gives rise primarily to neural tube although it also contains mesoderm precursors). *FGF8* is not just a marker of the caudal precursor region but it is also a crucial player in the regulation of cell maturation within its domain of influence. Cells with active FGF signaling pathway remain undifferentiated, both in the neural plate and in the mesoderm, while those with low or no activation of FGF signaling can progress to a more differentiated state (if the right signals are present) [17], [18]. It is therefore important to understand how this signaling pathway is regulated and in particular how the production of *FGF8* at both the protein and *mRNA* levels is controlled.

Some aspects of the regulation of *FGF8* expression are known. *FGF8 mRNA* is characterized by high stability so that cells that have stopped transcription of the gene can maintain its expression for a considerable time interval resulting in a graded distribution of the RNA in the extending axis [15]. Figure 1 illustrates that although *FGF8* transcription takes place in the CNPR, the area where *FGF8 mRNA* is present extends further rostrally. *FGF8* levels can be down-regulated by retinoic acid (RA) that is secreted from somites and this could in theory be sufficient for the progressive down-regulation of *FGF8* [17]. However, in the absence of RA, *FGF8 mRNA* is still progressively down-regulated [17] although its region of expression is expanded.

Although mechanisms responsible for the control of *FGF8* transcription remain unknown, it is clear that they must be coupled to caudal extension of the embryonic axis, a crucial process that takes place as *FGF8* is down-regulated. Caudal extension involves movements in all three embryonic layers that rely on different cellular behaviors that are region and embryo dependent. Many efforts have been made to understand the mechanisms that regulate convergence and extension of the mesodermal layer in fish and frogs where region-specific cellular behavior such as directed migration towards the midline (due to cellular intercalation) have been described [19]. More recently, a random cell motility gradient has been observed in chick presomitic mesoderm that contributes to axial elongation [20]. In addition, other phenomena such as stem-cell like mode of growth [21] reviewed in [16] and active movement of cells towards the caudal end [22] have been identified for neural plate and notocord elongation respectively. Extension of the embryo constitutes, therefore, a multi-factorial process where all these aspects of cell behavior are coordinated [23].

In this paper we will focus on two main features of vertebrate embryonic axis extension, namely progressive generation of cells not producing *FGF*8 and migration of the caudal precursor zone. We will use mathematical methods to analyze these processes.

Concentration dynamics of FGF8 and RA during caudal extension in chick embryo have been modeled previously in [24]. It was shown there that the dynamics of the concentration profiles of FGF8 and RA could, in theory, be explained by specific interactions (mutual inhibition) between FGF8 and RA which can be described by the system of nonlinear partial-differential equations having a propagating front solution.

The ability of local self-enhancement and long-ranging inhibition of morphogen gradients to give rise to a propagating front-like behavior has also been addressed in [25], where it was suggested that stationary patterns (Turing) form due to the growth of the medium (tissue grow due to cell proliferation).

The main feature of our approach is that we take into account the movement of the *FGF8* production domain and consider its effect on the dynamics of the *FGF8* concentration profile, as well as the effect of *FGF8* concentration profile on the differentiation and movement of cells. We perform our study using two distinct models. First, we develop and consider different modifications of a continuous one-dimensional model to check hypotheses concerning dynamics of morphogens and mechanisms of cell motion. Then we verify the obtained results by use of a multi-cell simulation method (the Glazier-Graner Hogeweg model, the *GGHM*, aka the Cellular Potts model or *CPM*) originally developed by Glazier and Graner [26], [27] and recently used to simulate and analyze the migration of cells in various biological tissues [28] including the formation of cell flows at the early stages of the chick embryo gastrulation [8].

Furthermore, with new experimental observations, we analyze modeling outcomes and further explore the mechanisms that underlie progressive down-regulation of *FGF8* and its role in the caudal precursor zone migration. We focus on the events that occur in the epiblast region that will give rise to the spinal cord (the CNPR) as this is a tissue where the regulation of *FGF8* transcription occurs but similar interactions may be relevant for mesoderm maturation. Based on our modeling and experimental results we suggest that the movement of the caudal precursor region is essential for the observed dynamics of the concentration patterns of involved morphogens, and that the interplay between these morphogens and the cells producing them is responsible for the progressive generation of differentiated cells as well as for the migration of the CNPR. We also show that the integration of cell proliferation, differentiation and movement allows the CNPR to maintain a constant size and preconditions the constant speed of its migration so that the moving stem zone regulates regression of the primitive streak. Table S1 outlines the summary of our models.

The mechanisms of embryonic axis extension we propose here may also be applicable to different systems where production of a morphogen by a domain of moving cells is responsible for progressive differentiation.

## Results

### Concentration profiles in the continuous one-dimensional model

We posed the general theoretical problem of what simple regulatory network could account for the restricted transcription of a gene within a domain of constant size considering that cells that transcribe the gene proliferate and move as the main axis of the embryo extends. To address this problem we first developed a continuous one-dimensional model (installation, templates for basic simulations and source codes are available from the web site: http://pcwww.liv.ac.uk/~mf0u4027/biochemsim.html). The simplest version of the model includes two variables: one for dynamics of hypothetical *A-*mRNA, transcribed exclusively in a fixed-sized domain that moves (say to the right) with constant speed (*c*), and one for the secreted protein it encodes (protein A). The basic model therefore considers concentrations of the following two species:

1. mRNA (non-diffusible) which is maintained (produced) at a constant level exclusively in a domain of constant size moving with a constant speed. Further on we will refer to the domain of mRNA transcription as to the DoT.
2. Protein A (diffusible) whose production rate is proportional to the level of *A-*mRNA.

**Figure 2:**
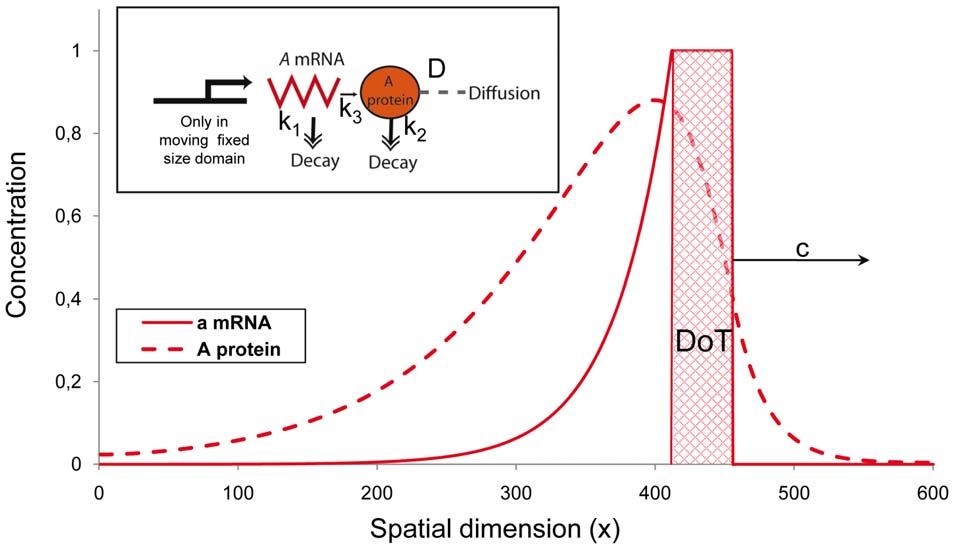
Stationary concentration profiles of *A-*mRNA and its corresponding protein in one-dimensional model of a migrating DoT. The solid red line denotes the concentration of *A-*mRNA along the embryo's axis while the dashed red line denotes the concentration of protein A. *A-*mRNA is produced in the DoT, i.e. in the red hatched area which has a preset size and moves to the right (the x-coordinate points to the posterior side) with speed *c*. Production of protein A is proportional to the level of *A-*mRNA. The schematic gene regulatory network diagram explaining the underlying molecular model is also presented. The detailed description of the model is given in the Materials and Methods Section. Here we presume that the DoT is located in the segment (420, 455) of the medium of total size 600 (space units) and moves with speed *c = 0.015* to the right. Other parameters: *k_1_ = 0.0003*, *k_2_ = 0.00025*, *k_3_ = 0.0005*, *D_2_ = 0.5*.

**Figure 3:**
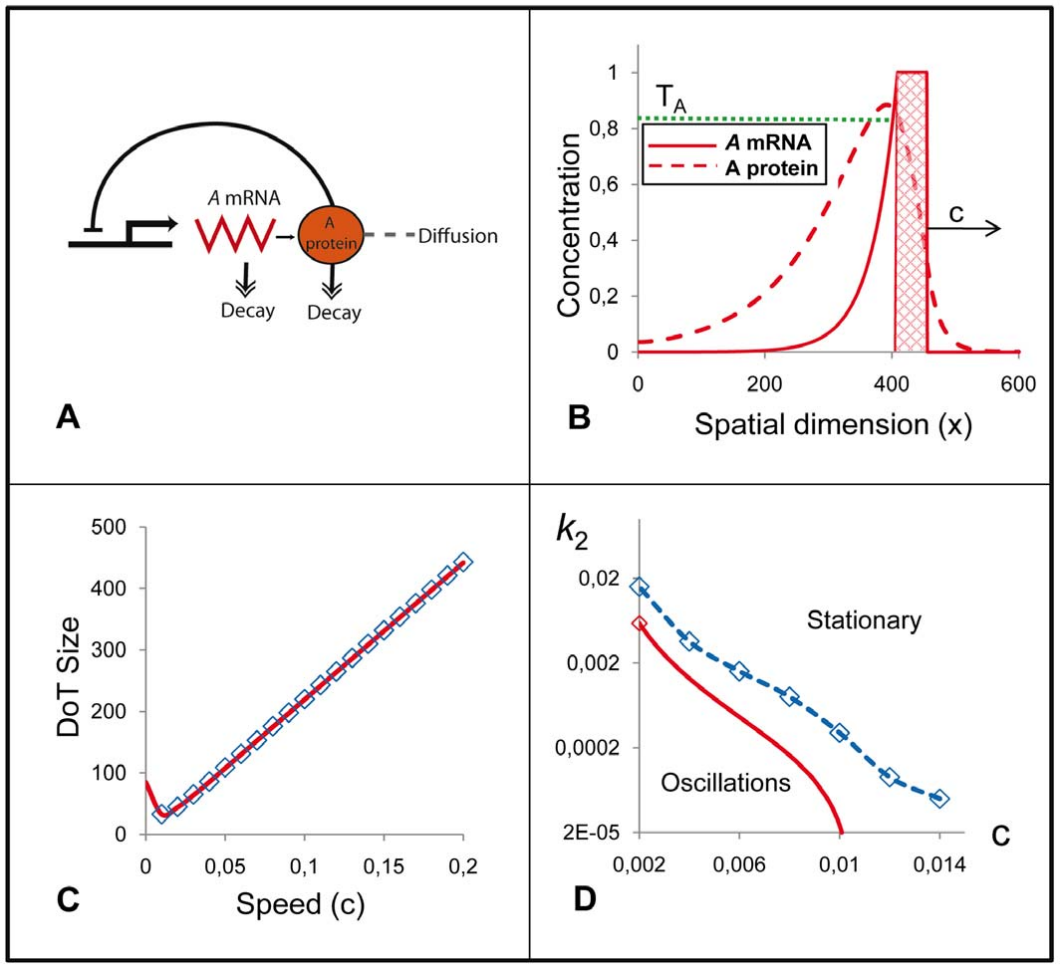
The model with cell differentiation. The basic model (Figure 2) is extended by imposing the condition that production of *A-*mRNA stops when the concentration of protein *A* reaches the threshold value *T_A_* (*T_A_ = 0.85* in all presented simulations). This defines the location of back (left) side of the moving DoT and therefore provides the mechanism controlling its size. **A**: The schematic gene regulatory network diagram explaining the used version of the model, for further details see the Materials and Methods Section. **B**: Concentration profiles of *A-*mRNA (solid red) and protein A (dashed red) with respect to the moving DoT (red hatched). Parameter values are the same as in Figure 2. **C**: The DoT size versus the DoT speed in simulations (blue markers) and in analytics (solid red line, given by the equation 9 in the Materials and Methods Section). **D**: Domains corresponding to the stationary and oscillating dynamics of the DoT size on the parameter plane *“k_2_ versus c”* in simulations (blue markers) and in analytics (red line). *c* is the velocity of the DoT migration and *k_2_* is the kinetics rate (1/*k_2_* is a relaxation time) of protein *A* (here and everywhere else *k_3_ = 2k_2_*).

Figure 2 shows the stationary concentration profiles of both species with the assumption that the decay rates are given by linear functional terms (see the description of 1D model in the Materials and Methods Section). The transition process from the initial conditions (when both concentrations are zero everywhere except for the DoT where the concentration of *A*-mRNA is one) to the stationary solution is shown in Movie S1. Also, since the DoT is moving, the concentration profiles of *A-*mRNA and protein A do not form symmetric pattern with respect to each other: *A-*mRNA decays gradually behind the DoT and the maximum in protein A profile lags behind (shifted to the left in the graph) the midpoint of the DoT. This shift becomes more pronounced with the increase of the DoT speed, *c*, and depends on the kinetic rates of *A-*mRNA and protein A (see equation 6, Matherial and Methods). Due to this shift the maximum in concentration of the protein can lie outside the DoT (see Figure 2, also Figure 3B). The condition for this is given by inequality 7 in the Materials and Methods Section.

### Self-regulation of the size of the DoT via negative feedback

So far in our model the size of the DoT (which reflects the number of cells transcribing *A-*mRNA) has been fixed. Now we would like to take into account that the cells forming the DoT proliferate and differentiate (i.e. can stop transcribing *A-*mRNA under the appropriate conditions). When proliferation is taken into account the DoT size should gradually increase over time unless a regulative mechanism ensures this does not occur. The shape of the concentration profile of protein A in Figure 2 gives an idea of a possible and simple mechanism for regulating the size of the DoT that would not involve any component external to the system. If we assume that cells stop transcription of *A*-mRNA when the level of protein A rises above some threshold, *T_A_*, (see the diagram on Figure 3A), this would define the position of the left side border of the DoT (as a coordinate of the point where the level of protein A gets above *T_A_*) while the position of the right side border is predefined and given as a coordinate of a point moving to the right with speed ***c*** (see Figure 3B). Now the size of the DoT is defined by the negative feedback loop where protein A inhibits the transcription of *A*-mRNA.

In this version of the model the size of the DoT is defined by the value of the threshold *T_A_*: when the concentration of the protein gets above *T_A_*, transcription of the *A-*mRNA stops and this eventually defines the DoT size. This mechanism of the DoT size regulation works if the value of parameter *T_A_* is below the maximum possible value of protein A concentration (which is *k_3_/k_2_*, see equations 2 and 4 in the Materials and Methods section). Obviously, the size of the DoT is small for low values of *T_A_* and increases with *T_A_*. Simulations and analysis of the model show that the size of the DoT is generally an increasing function of the DoT's speed (see Figure 3C). Simulations also indicate that this size is not necessarily a constant and can oscillate over time (compare Movies S2 and S3 showing formation of a DoT of stationary and oscillating sizes). Oscillations are observed when the kinetics rate of *A-*mRNA (*k_1_*) or protein A (note that for simplicity we presume that *k_3_ = 2k_2_* in all simulations, i.e. the rates of protein A production and decay are varied in a proportional manner) are too small with the transition (bifurcation) value depending on the speed of migration of the active transcription domain (Figure 3D). Comparison of the numerical and analytical results indicates that the domain in the model parameter space, where oscillations are observed numerically, strongly correlates when no stationary solution exists according to analytics (compare dashed blue and solid red lines in Figure 3D and see inequality (10) in the Materials and Methods Section).

### Size regulation of the FGF8 domain of transcription

So far, we have kept our model general and have not named the molecules that would be represented as morphogen A. Going back to the CNPR, we are interested in understanding the mechanisms that regulate the size of the *FGF8* transcription domain and therefore the CNPR. The simplest possibility would be that FGF8 corresponds to morphogen A. In this case, based on the results presented in Figure 3, we could suggest that high FGF8 levels stop transcription of its own gene and thus regulates the size of its domain of transcription.

**Figure 4:**
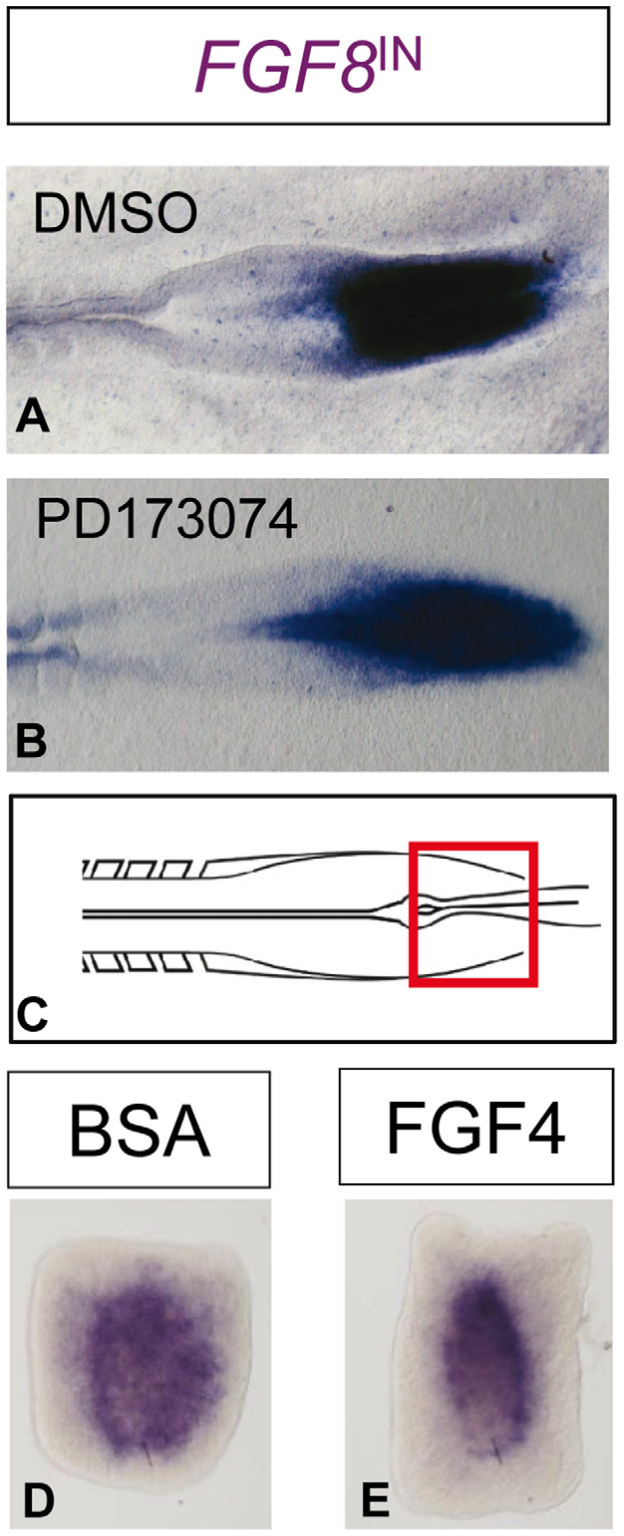
*FGF8* transcription is not altered by FGF signaling. **A–B:** *FGF8* transcription at the caudal precursor zone in control (**A**) and FGFR antagonist treated (**B**) chick embryos. No changes in *FGF8* transcription are observed following a blockade of FGF signaling. **C:** Schematic drawing showing the origin of the explants shown in **D–E**. **D–E:** *FGF8* transcription in caudal precursor zone chick explants following culture in the presence of control (**D**) and FGF4 containing media (**E**).

Previous reports suggest that FGF8 may be able to promote the stability of its own mRNA transcript [15], but no experimental evidence for its influence on the rate of its own transcription has been found. In order to examine the dependence of *FGF8* transcription on FGFR signaling, we treated chick embryos with the FGFR antagonist PD173074 for 4 h [29]. This treatment did not change significantly the domain of *FGF8* transcription (compare panels **A** and **B** in Figure 4) (n = 6). In addition, the treatment of cultures of the caudal precursor zone with FGF4 (which activates FGF receptors more efficiently than FGF8 [30]) for 9 h did not alter *FGF8* transcription (n = 3, Figure 4 panels D and E). These results in the chick embryo are consistent with the phenotype of *FGFR1* mutant mouse embryos where the expression of *FGF8* in the caudal precursor region is not altered [31]. Therefore we conclude that *FGF8* is not self- repressing.

**Figure 5:**
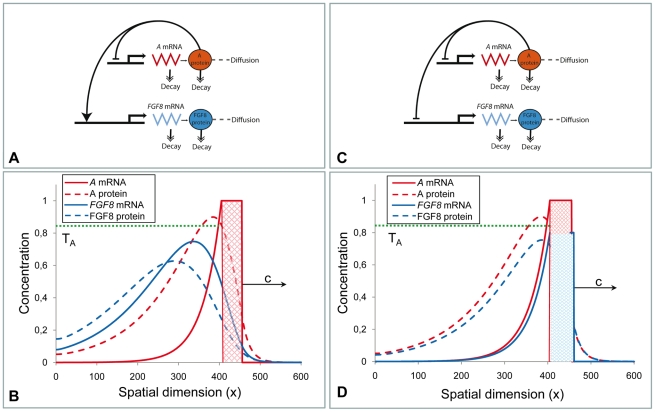
Possible mechanisms of the involvement of *FGF8* in the caudal gene regulatory network. **A–B:** the rate of *FGF8* transcription is proportional to the level of protein A (see the Materials and Methods Section for details). Note that the *FGF8* DoT extends behind the *A-*mRNA DoT. **C–D:** the transcription of *FGF8* and *A-*mRNA are launched independently in (roughly) the same group of cells while both down-regulated by the same signal provided by protein *A*. Note, that the concentration profiles for *FGF8* DoT and *A-*mRNA DoT in this case basically coincide. Values of parameters (in equations 1 and 2) are the same as in Figure 2. For extra parameters (equations 11 and 12): *D_4_ = 0.5*, *k_31_ = k_41_ = 0.0003*, *k_32_ = k_42_ = 2k_31_*.

In terms of our model this means that FGF8 dynamics could be regulated by the caudal self-repressing morphogen A. We have explored several possible relationships between this self-repressing morphogen (protein A) and *FGF8*. If *FGF8* transcription was activated by protein A then the profile of *FGF8* transcription (and therefore the extent of the CNPR) would lag behind the domain of the *A-*mRNA transcription (Figure 5A–B). Alternatively, if the transcription of *A-*mRNA and *FGF8* is initiated in a similar caudal domain and protein A is repressing simultaneously the transcription of both, then the DoT of *FGF8* would coincide with (or at least will not significantly differ from) the DoT of *A-*mRNA (Figure 5 C–D). Both possibilities are feasible in principle but for simplicity, in the following sections, we will consider the latter option, where the domains and concentration profiles of morphogen A and *FGF8* are equivalent.

### Maintenance of the migrating DoT size in GGHM

We have explored some features of the migrating DoT by means of a continuous 1D model. However this model does not provide the most appropriate framework to model cells that are proliferating and moving and therefore we have extended our study by developing and using an individual-cell based model represented by Glazier-Graner-Hogeweg Model (GGHM) [28]. This modeling approach allows us to test more carefully the phenomena emerging from the individual cell behaviors.

The epiblast caudal precursor region in the chick embryo consists of a unicellular layer of cells and therefore we can use the 2D version of the GGHM to capture events taking place over the CNPR (here we are not dealing with the influence of external signals coming, for example, from the mesoderm). Installation of the program and templates for reproduction of our simulations are available from http://pcwww.liv.ac.uk/~mf0u4027/biocellsim.html.

**Figure 6:**
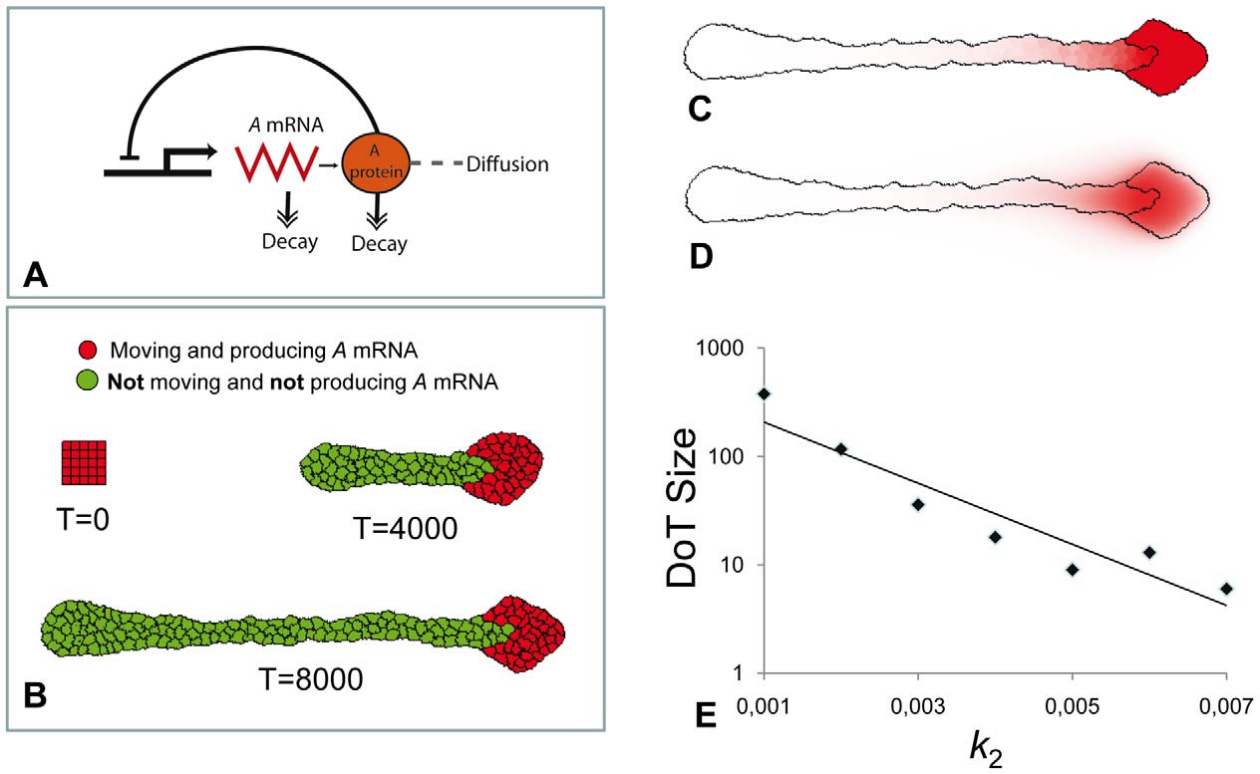
The DoT migration in the GGHM. **A:** Schematic diagram of the used model (identical to the diagram in Figure 3). **B:** Three consecutive images from the simulation of primitive streak regression. Initially there is a group of 25 red cells (the DoT) forming a square tissue. The level of *A-*RNA is high and constant in all red cells. Red cells move (to the right), proliferate and differentiate, i.e. red cell transforms into the green cell when the level of protein *A* at any point inside the red cell gets above the threshold value *T_A_ = 0.8*. Green cells do not move nor produce *A-mRNA*, for simplicity we have also assumed that they do not proliferate. Cell differentiation is regulated by the level of morphogen *A* (as in Figure 3). Parameters: *k_1_ = 0.001*, *k_2_ = 0.003*, *β = 4.5*. **C**, **D:** Concentrations of *A-*mRNA (**C**) and protein A (**D**) are shown in shades of red. The border line between red and green zones is along an isoline in the concentration field of protein A corresponding to the threshold value *T_A_ = 0.8*. **E:** Increase in the rate of *A* kinetics, *k_2_*, (assuming that *k_3_ = 2k_2_*) reduces the size of the DoT (or number of cells forming the DoT) exponentially.

The version of the GGHM, which corresponds to the 1D model used above (see Figure 3), includes two cell types only (Figures 6A–B): cells transcribing *A-*mRNA (red) and cells which do not transcribe it (green). The dynamics of *A-*mRNA and protein A are defined the same way as in the 1D model except for: (a) equations 1 and 2 (see Materials and Methods Section) are written for the laboratory frame of reference (*c = 0*) and (b) diffusion term in equation 2 is extended into 2D. To be in line with the differentiation mechanism suggested for 1D model we assume that the red cells proliferate and convert into green cells when the level of protein A reaches the threshold value, *T_A_*. We also attribute motility properties to cells, namely, we presume that red cells move actively while green cells do not and can only follow red cells due to adhesive contacts.

We start the simulation with a group of red cells (the DoT) moving in a particular direction (to the right in Figure 6B) under the influence of a preset force. This force is given by the extra term *E_f_ = −β_f_ (* ***x·i*** *)* (where ***x*** - shift of red cell's interface and ***i*** – the unit vector pointing to the right) in the definition of the energy which counts for the work done by the horizontal force applied to moving cells. This permits us to leave the study of the mechanisms of cell motion for later (see below). While moving and proliferating, cells in the DoT transcribe *A-*mRNA which in turn allows the production of protein A (see Figures 6C and D). In places where the level of protein A reaches its threshold level, *T_A_*, red cells differentiate into green. Simulations show that, while moving and proliferating, red cells (forming the DoT) leave a trail of differentiated green daughter cells (Figure 6B and Movie S4) very similar to what happens in the embryo where the CNPR gives rise to more mature tissue progressively. The size of the DoT (number of red cells) is regulated by the kinetics of both *A-*RNA and protein A: increasing either kinetics constant k_1_ or k_2_ (assuming for the latter that *k_3_ = 2k_2_*) decreases the size (area) of the DoT (see Figure 6E).

### Promotion of cell migration by a caudal morphogen

The simulations presented in Figures 2, 3, 5 and 6 were performed under the assumption that the ability of cells to move correlates strongly with their ability to transcribe the *A-*mRNA gene so that the high level of protein A switches off both abilities of a cell. However other mechanisms that relate motility of cells to morphogen concentration may fit better to experimental results and known signaling molecules produced in the caudal precursor zone, in particular FGF8. Our 2D (GGHM) model can be used to check some of these mechanisms.

In our model we were dealing with a hypothetical protein A rather than *FGF8*, but as we have previously explained, if protein A down-regulates the transcription of both *A-*mRNA and *FGF8*, the concentration profile of protein A is equivalent to that of FGF8. The signaling pathways that regulate the movement of cells in the caudal precursor zone are not well established, although it is known that FGF signaling controls the ability of spinal cord precursor cells to move [32]. Down-regulation of FGFR signaling in one cell promotes its exit from the CNPR which suggests that FGF signaling keeps cells moving and allows them to accompany the regressing Hensen's node [32].

**Figure 7:**
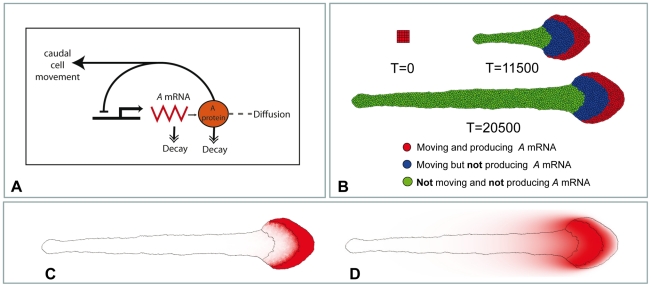
The DoT migration in the GGHM with 3 cell types. **A:** Schematic diagram of the used model. **B:** Snapshots from the simulation: red cells – move and produce *A-*mRNA, blue cells move but don't produce *A-mRNA* and green cells do not move and do not produce *A-mRNA*. Red cells transform into blue when the level of protein *A* rises above *T_A_ = 0.75*, blue cells – to green when the level of protein *A* drops below *T_M_ = 0.6*. Only red cells proliferate. Parameters: *k_1_ = 10^−3^*, *k_2_ = 5·10^−4^*, *β = 3.8*. **C**, **D:** concentration profiles of *A-*mRNA (**C**) and protein A (**D**) after 20500 time steps of simulation.

To incorporate this feature into our model we decouple the ability of cells to transcribe *A-*mRNA from their ability to move and we introduce an intermediate cell type: cells that do not transcribe *A-*mRNA but can move (blue cells in Figure 7). Therefore differentiation of red cells into green cells takes place in two steps. Step 1: we assume that transcription of *A-*mRNA is down-regulated by protein A, i.e. production is stopped when the level of protein A achieves some threshold level *T_A_* (analogous to 1D model represented in Figures 3A and 5C). Step 2: all cells transcribing *A-*mRNA move and those that do not transcribe *A-*mRNA keep moving until the level of protein A gets below another threshold *T_M_* (*T_M_*<*T_A_*). The shape of tissue formed by cells forming the DoT and their descendants (all daughter cells) is given on Figure 7B (see also Movie S5). Comparing Figure 6B and Figure 7B we can see that both modifications of the model allow regulation (stabilization) of the DoT size.

### Chemotactic mechanism for the DoT migration

Up to now we were presuming that the DoT is moving and that the speed of its motion is given by the preset parameter *c*. Examination of Figure 3 reveals a possible mechanism of this motion. Assume that the cells forming the DoT are chemotactic to a morphogen they produce. For example, we can assume that the speed of the DoT migration is proportional to the gradient of A: 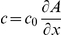. The gradient can be taken at some specific point, for example at the right border of the DoT, or we could use an average gradient over the entire DoT, i.e. the difference between concentrations of the protein A on two borders of the DoT divided by the size of the DoT. Computer simulations show that both these assumptions can cause the DoT migration with constant speed and therefore the motion of the CNPR can have a chemotactic nature. Simulations with the first assumption, i.e. the speed of the DoT is defined by the gradient of A on one particular side, show that starting from a wide range of initial conditions (and also for a wide range of values of *c_0_*), we obtain (after some transition period) a DoT migrating moving with constant speed (see Movie S6). Simulations with the second assumption, i.e. that the speed is defined by the average gradient of morphogen A over the DoT, also show the desired behavior, but we need to apply special initial conditions: for example, we force the DoT to move for some initial time and then switch this force off and chemotaxis on (see Movie S7).

**Figure 8:**
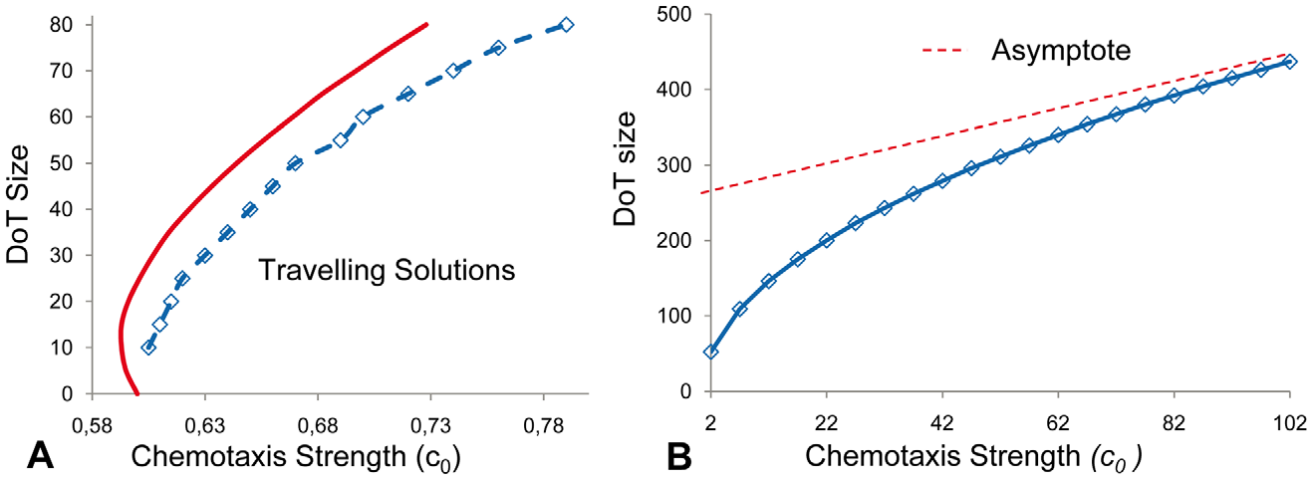
The DoT migration due to chemotaxis in 1D model. The speed of the DoT migration is defined by the formula *c = c_0_(A_l_−A_r_)* where *A_l_* and *A_r_* are concentrations of protein A on the left and right borders of the DOT. **A:** The version of the model where the DoT size is fixed (chemotaxis without *A*-RNA self-repressive production control). The domain in the parameter plane “*a versus c_0_*” where the travelling DoT should definitely exist according to the analysis of the model is on the right side of the solid red line (this line represents the border of the domain defined by inequality 16 in the Materials and Methods section. Transition points (between existence and nonexistence) of migrating DoTs in simulations are given by blue markers, dished blue line connecting these markers gives the numerically obtained border. **B:** The version of the model where the DoT size is controlled by the protein A (chemotaxis with *A*-mRNA self-repressive production control). The size of the DoT depends on the parameter *c_0_*. The difference *A_l_−A_r_* depends on *c_0_* and saturates when *c_0_→∞* giving a linear asymptotic (red) for the dependence of the DoT size on *c_0_*.

Thus the migration of the DoT can be due, in theory, to chemo-repulsion of its constituent cells by protein A. This mechanism of migration is very similar to that of so called “ballistic” motion of a point which is a source of its own chemo-repellent [33]. In our case this “ballistic” effect is even more profound: the chemo-repellent is produced not only inside the DoT but also behind it (where the concentration of *A-*mRNA is nonzero) and this adds to the difference between concentrations of the chemo-repellent at the front and back sides of the DoT. Simulations as well as analytical studies of the model show that the migration (with constant speed) of a self-repelled DoT is only possible when the parameter *c_0_*, defining the strength of chemotaxis, is above a certain threshold (see Figure 8A, where this threshold is roughly 0.6). The concentration profiles of *A-*mRNA and protein A, as well as the size of the DoT, depend on the parameter *c_0_* similar to their dependences on the parameter *c* in the non-chemotactic version of the model (equations 8–10) with fixed speed of the DoT migration (compare Figure 3C with Figure 8B).

The role of chemotaxis in the migration of caudal precursor zone has not yet been addressed experimentally, but it is known that FGF8 can act as a chemo-repellent upon mesenchyme cells during gastrulation in the chick embryo [9], and that down-regulation of FGF signaling does not allow the caudal movement of cells following node regression [32]. Although, in our model, we consider the self-repressing morphogen A as the chemo-repellent for cells forming the DoT, the same result would be obtained if we consider that the actual repellent is FGF8 whose dynamics coincides with that of protein A (as discussed above).

### Chemorepulsion in GGHM

As we have noted in the previous section, the migration of the DoT can have a chemotactic nature and, in addition, there is strong evidence that FGF8 can act as a chemorepellent in several contexts [34]. Using GGHM we can analyze this problem to a much greater extent than was possible in the framework of the 1D model.

**Figure 9:**
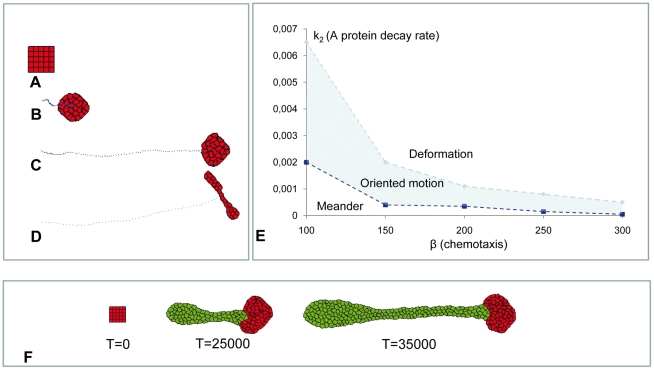
Migration of the DoT due to chemotaxis in GGHM. **A–E:** Simulation of the movement of a group of cells transcribing *A-*mRNA (DoT) that are repelled by a protein A they produce. Three types of behavior can be found in GGHM. Here we assume that cells forming the DoT do not grow, proliferate or differentiate. **A:** Initial shape of the DoT. The DoT was “forced” to move to the right (see about a preset motion of the DoT in Figure 7) for the first 2000 time steps to provide initial conditions for chemotactic motion. “Self-repelled” DoT can: **B:** meander (*k_2_ = 0.0005*). **C:** move along straight line (migrate) (*k_2_ = 0.0025*). **D:** move and elongate (deform into “umbrella”-shaped tissue) (*k_2_ = 0.0055*). **E:** domains on a parameter plane (*k_2_ versus β*) corresponding to each kind of behavior. Blue dots in **B**, **C** and **D** show location of the DoT's center of mass every 200 time steps. Parameters: *k_1_ = 0.001*, *β = 120*. **F:** Simulation of the movement of a group of red cells transcribing *A-*mRNA (DoT) that are chemotactically repelled by a protein A they produce and that, in addition, grow, proliferate and differentiate into green non-actively moving cells (as it was in the case of Figure 6). Initially the DoT is represented by a group of 25 cells. These cells are “forced” to move to the right for the first 2000 time step computations to provide the direction for further chemotactic movement. After *T = 2000*, red cells are repelled by protein A, and (as directed by the initial conditions) they move to the right leaving the trail of differentiated daughter cells (green).

Let us first consider a simplified problem by ignoring cell proliferation and differentiation. Assume that the DoT is represented by a group of (non-proliferating) cells which produce some chemotactic agent (protein A or FGF8, in our case), such that the cells are repelled by this agent. Is it possible that this group of cells will migrate (move along a straight line) due to this repulsion and thus reproduce migration of the DoT? Our simulations show that the group of cells repelled by a chemical they produce can exhibit three types of behavior (Figure 9). Cells can stay as a compact group and move randomly or meander with little net relocation (Figure 9B, Movies S8) or exhibit oriented motion, as in the case of the CNPR (Figure 9C, Movie S9). Movement of cells can deform the shape of the DoT (Figure 9D, Movie S10) or even break it so that they form a few smaller groups of cells each moving independently. The type of observed behavior is defined by model parameters and can be altered by varying the number of cells, their adhesiveness (defined by the adhesion matrix *J*), kinetics rates of protein A (production *k_3_* and decay *k_2_*), *A-*mRNA (decay *k_1_*) and chemotactic forcing, β, (see Materials and Methods Section). Generally, the model's parameter space can be represented as a collection of domains corresponding to each type of observed dynamic behavior. Figure 9E shows the location of these domains on a parametric plane corresponding to two key parameters (responsible for the type of dynamics exhibited by self-repelling group of cells), namely chemotactic force as defined by parameter *β* and protein A decay rate, *k_2_* (see Materials and Methods Section). When the chemotactic forcing is weak (*β* is less than some threshold value, and this threshold depends on *k_2_*) the group of cells meanders and shows no net migration. The meandering behavior is intrinsic to GGHM (corresponds to thermal fluctuations when *T>0*) and it is a counterpart of the resting DoT in continuous 1D model: as we saw previously the DoT in 1D model does not migrate (or no traveling solutions exist) when *c_0_* is below than some threshold value (see inequality (16)) and this threshold depends on the DoT size (see Figure 8A). A meandering group of cells starts to move along a straight line when we increase the chemotactic forcing (by increasing the parameter *β*) or the protein A decay rate, *k_2_*. This type of behavior is also in a line with our observations on 1D model where the DoT starts to migrate when chemotactic forcing *c_0_* is above some threshold value (Figure 8A). On the other hand further increase in either of these parameters results in deformation of moving tissue so that rounded tissue transforms into an umbrella-like shape. There is no 1D counterpart for this kind of behavior.

Thus, the migration of the *A-*mRNA DoT can be explained by the chemotactic response of its constituent cells to protein A (i.e. the *FGF8* DoT migrates due to repulsion by *FGF8*). Now we can put proliferation and differentiation of cells back into this model and adjust model parameters so that we observe oriented motion of tightly packed red cells leaving the trail of differentiated daughter cells (Figure 9F, see also Movie S11). The result from this simulation mimics the regression of the CNPR indicating that the interactions we have considered are sufficient to account for the observed maintenance of a compact group of cells that proliferate, migrate and differentiate during vertebrate embryonic axis extension.

### Experimental study of regulative properties of the FGF8 DoT

**Figure 10:**
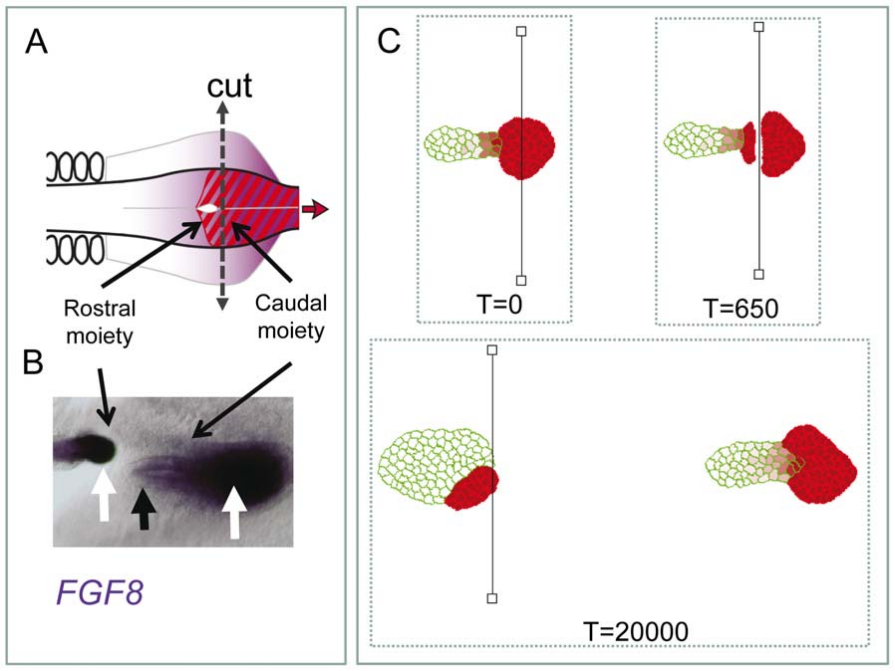
Regulative properties of the FGF8 DoT. *FGF8* expression can be maintained in the absence of caudal-most signals and can be progressively down-regulated in the absence of rostral signals. **A:** Schematic diagram showing the experimental separation of the rostral and caudal parts of the FGF8 DoT (cutting experiment). **B:** *FGF8* expression following the experimental separation of the caudal precursor zone into two. White arrows show how *FGF8* is maintained at both the rostral and caudal moieties and black arrow shows the progressive down-regulation of *FGF8* in the caudal moiety. **C:** Simulation of cutting experiment: the chemotactically moving DoT is cut into two pieces (see images at *T = 0* and *T = 650*). We allow the piece corresponding to the caudal moiety to move while the movement in the rostral moiety is arrested by the cut. The concentration of *A-*mRNA (shown by shades) which is associated with the location of the DoT reproduces the corresponding pattern for *FGF8* shown in **B**. Parameters: *k_1_ = 5·10^−4^*, *k_2_ = 2.5·10^−5^*, *β = 1950*.

In order to challenge the ability of the model to reproduce experimental results, we have performed an experiment where the *FGF8* DoT (which in our model is equivalent to the *A-*mRNA DoT) was split into two and the changes in the expression of *FGF8* were analyzed after 20 h culture (Figure 10A, B). In the rostral moiety, *FGF8* was maintained caudally suggesting that *FGF8* does not require signals from the caudal-most region of the embryo for its maintenance. In addition, this experiment also shows that the capacity of *FGF8* to progressively down-regulate is also intrinsic to the caudal moiety (Figure 10B).

Using our complete model, we have performed simulations where we split the DoT into two (Movie S12) and follow the behavior of the two moieties. As shown in Figure 10C we find the maintenance of a caudal population of red cells (those producing *A*-RNA or the equivalent *FGF8*-RNA) in the rostral moiety and the progressive generation of a green population (that have stopped producing *A*-RNA) in the caudal moiety, very similar to what is observed in experiments (Figures 10A, B).

This result suggest that our model captures essential features of the biological network regulating *FGF8* expression and encourage the search for a morphogen A with both the ability to self-repress transcription of its encoding RNA and of *FGF8*. In addition, it posses the possibility that chemotaxis may play a role in caudal elongation of the embryo.

## Discussion

The aim of this work is to explore possible mechanisms of progressive differentiation and regression of the caudal neural precursor region (CNPR, as defined by the region where cells transcribe *FGF8* mRNA) in the chick embryo. We have focused on essential features of regression of this region such as progressive differentiation and the conservation of its size and speed of migration. These features were incorporated into two distinct modeling approaches which we used to evaluate a set of hypotheses concerning the mechanisms of differentiation and motion of cells in the CNPR. Our results are summarized in the Table S1.

The regulation of FGF8 dynamics during the regression of the caudal precursor zone was previously addressed using a mathematical model [24] where it was suggested that a mechanism involving FGF8 self activation could, in theory, account for the progressive down-regulation of FGF8 provided a high FGF8 degradation rate was considered. According to this model the dynamics of FGF8 can be described as a propagating concentration wave, which is one of the patterns that form in nonlinear reaction-diffusion systems [35].

In the present work we have also addressed the regulation of FGF8 dynamics but we consider both intracellular (mRNA) and diffusing (protein) species of *FGF8* and most importantly we take into account the movement of the domain of *FGF8* transcription.

Cells in the precursor region proliferate and differentiate in such a way that the size of the region (identified by *FGF8* transcription) does not change significantly over time. The size of a growing tissue usually involves control of proliferation such that when the tissue reaches a certain size, cells stop dividing. This may involve a mechanism which is able to measure population size as has been described in bacteria quorum sensing in *Dictyostelium* population, the *Drosophila* imaginal disc [36] or for the mesoderm community effect [37]. It is generally hypothesized that the concentration of signaling factors change as the size of the tissue increases until they reach a threshold value that dictates an arrest in cell proliferation. In our scenario, maintenance of a population with constant size is not due to an arrest of proliferation but to the balance of proliferation versus differentiation that is spatially controlled such that only cells at the rostral end of the domain differentiate (i.e. stop transcribing *FGF8*). This involves a mechanism where the strength of the signal regulating cell differentiation correlates with the size of the cell population, i.e. the signal is provided by a morphogen whose overall production is related to the size of the zone. In terms of our model this could be morphogen A produced from *A-*mRNA which in turn is produced exclusively in the precursor region. This is reflected by our model assumption that cell differentiation takes place when the level of morphogen A rises above some threshold, *T_A_*. This assumption allows the control of the DoT size, although (depending on parameter values) the size can be stationary or oscillating (see Figure 3). An interesting problem is whether the DoT size is stationary or oscillating in experimental conditions.

Another important problem is what morphogen is actually under self-repression control and can be involved in the regulation of the DoT size. One possibility would be that *FGF8* is actually able to repress transcription of *FGF8* mRNA, however this is not supported by our experimental evidence as manipulations of the level of *FGFR* activation in experimental conditions do not seem to affect the size of the area where *FGF8* mRNA is expressed. This brings us to an alternative assumption that, for example, another morphogen is responsible for the regulation of *FGF8* transcription. Two possibilities have been considered: morphogen A activates *FGF8* transcription or it represses *FGF8* transcription (see Figure 5). Both are able to maintain a domain of *FGF8* transcription of constant size; however the latter network would account more easily for the maintenance of *FGF8* expression in the rostral fragment following the splitting of its domain of expression.

Several secreted proteins are present in the caudal zone that could correspond to *A-*mRNA such as *WNT*s (*WNT3A*, *WNT8C*) and *BMP*s (*BMP7*, *BMP4*). They could participate in the mechanisms presented in Figure 5. Independently of the particular mechanism that regulates production of *FGF8* in our models, the relevant feature of the regulatory networks that allows the maintenance of a constant size of the domain transcribing *A-*mRNA is the presence of a negative feed-back loop involving protein *A*.

It is known that retinoid acid signaling from the somites is involved in down-regulation of *FGF8*: in the absence of RA the domain of *FGF8* is expanded. However, down-regulation of *FGF8* still occurs in RA-deficient embryos and our experiments of embryo sectioning show that progressive down-regulation can occur in the absence of rostral signals. In our model we did not take into account the influence of the rostro/caudal gradient of RA in shaping the *FGF8* pattern. Future work will be required to incorporate into the models more elements concerning the gene regulatory network involved in *FGF8* regulation such as the influence of RA, which is itself influenced by FGF signaling and Wnt8C, which is regulated by RA and FGF8 [6], [38].

Our models assume the existence of concentration thresholds of morphogen A that determine whether *A*-RNA (or *FGF8*) is transcribed or not. Several molecular mechanism underlying such an all-or-nothing response of cells could be relevant in this context, such as nonlinear saturating autocatalytic feedback of a gene product [39] or mutual inhibition [25]. It has been suggested that mutual inhibition of FGF8 and RA gradients may be involved in setting a bistability switch of FGFR versus Retinoic acid receptor activation. However so far, no experimental evidence indicates that such a switch could be involved in controlling whether *FGF8* is transcribed or not [40].

Coordination of differentiation and axis extension can be found during growth of plant meristemes and in vertebrate limb bud development. In these cases, however, the mechanism involved must be different to caudal extension as differentiation coupled to axis extension relies on an external cell population that secretes a morphogen that regulates proliferation and maintains neighboring cells in an undifferentiated state. In the case of the root meristeme this is the quiescent center, in the case of the apical shoot meristeme it is the organizing center [3], [4] and in the case of the limb it is the apical ectodermal ridge that secretes FGFs [5].

The other feature that we have explored using our models is the mechanism of domain migration. Several cellular behaviors have been shown to contribute to regression of the primitive streak-node and extension of the embryo. Convergence (at the midline) and extension seem to be at play in mesoderm. Besides, stem-cell like mode of growth and caudal movement of cells have also been observed in the neural tube and axial mesoderm [32], [41]. At the caudal neural plate, FGF signaling is required for cells to accompany the regressing primitive streak and precocious down-regulation of the pathway results in cells exiting the node-streak region. The version of the GGHM with differentiation (incorporating the influence of FGF8 on *FGF8* transcription and cell motility) shows that such a mechanism is able to maintain a cohesive group of cells moving at constant speed (Figure 6). Further extension of the model with the assumption that the reason why cells move caudally is related to FGF8 concentration (FGF8 acts as a chemorrepelent) allows us to simulate the correct behavior of cells that can move coherently in one direction provided there is an initial cause for the migration. A stationary group of cells producing a chemotactic agent maintains a symmetric condition with respect to the agent's concentration profile and will not move unless other events (such as noise) are involved. Indeed, Hensen's node (which we considered here as a part of stem zone) changes direction of its motion when the progression of primitive streak is replaced by its regression. We don't know what is responsible for reversing the motion of Hensen's node but most likely it is due to some external signals, while the repulsion by morphogen A in our model is rather an internal process as the production of this morphogen is closely associated with the processes in the caudal precursor region itself.

In summary, we have used mathematical models to explore possible mechanisms for the progressive differentiation of the caudal stem zone coordinated with the embryonic rostro-caudal extension. We have found that the self-repression of a caudal morphogen could be involved in driving progressive differentiation of the caudal stem zone and that chemo-repulsion here may be part of the mechanism responsible for the axis extension. Further experimental evidence is required to assess the role of FGF in regulating motility of ectodermal cells and to find out the signaling pathways that may be at the core of these mechanisms.

## Materials and Methods

In this section we describe the mathematical models as well as the experimental techniques used for obtaining the results presented in this work. For our study we have developed two models: continuous (1D) and cell-based (2D, Glazier-Graner Hogeweg model also known as Cellular Potts model). Dynamics of morphogens was modeled in the same way in both models while the migration of the DoT - using different techniques. In the 2D model we have considered a tissue consisting of a single layered group of cells. Each cell can produce and/or degrade genes and proteins and, in addition, move in response to the forces (adhesive, chemotactic) acting upon it. Also, the 2D model incorporates the ability of cells to grow and proliferate.

### One-dimensional continuous model

1D simulations were performed in a medium of fixed size in a frame of reference moving with the DoT. To describe the dynamics of morphogens we have used reaction-diffusion equations with an added advection term to take into account the DoT migration.

#### Basic model

The basic model is represented by two equations: one – for the dynamics of the concentration of a non-diffusible agent which we call *A-*mRNA and the second – for the concentration dynamics of corresponding protein *A*. The concentration of *A-*mRNA (denoted as *u_1_*) is equal to 1 (i.e. constant) inside the DoT of fixed size, *a*, while outside is given by the equation:
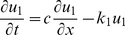
(1)


Parameter *k_1_* defines the rate of *A-*mRNA decay while parameter *c* defines the speed of DoT migration or the speed of the frame of reference. The concentration of protein A (denoted as *u_2_*) is defined by the equation:
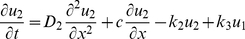
(2)where parameter *D_2_* defines its diffusion constant while *k_2_* and *k_3_* - the rates of protein decay and production. Production of the protein A is assumed to be proportional to the concentration of *A-*mRNA while its decay is proportional to its own concentration. The stationary solution of the system (1–2) can be found analytically. One can check directly that the solution:
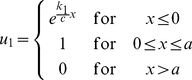
(3)satisfies (1) while the solution:
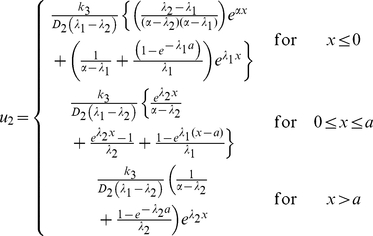
(4)where
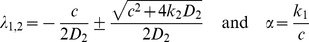
(5)satisfies (2). Figure 2 shows typical concentration profiles given by (3–4).

Since the DoT is moving, the maximum of the concentration of protein A lags behind the middle of the DoT, i.e. *x_max_<0.5a* where *x_max_* is the location of the maximum. For a slowly moving DoT the maximum is located inside the DoT (*0<x_max_<0.5a*) with its coordinate defined by the condition that the derivative of the *u_2_*-solution inside the DoT, *0≤x≤a* see (4), is zero. This gives:
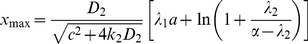
(6)This coordinate is *a/2* when *c = 0* and decreases with the increase of c. When the DoT's speed is too high the maximum lags behind the DoT, i.e. *x_max_<0*. The condition for this case can be given, for example, by the following inequality:
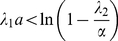
(7)when *x_max_* defined by (6) becomes negative. This condition is also confirmed by consideration of the maximum for *u_2_*-solution behind the DoT (*x≤0* in (4)).

#### Model for the regulation of the DoT size

In order to consider the proliferation and differentiation of cells in the DoT we extend the basic model by the assumption that the location of the left side (or back side in respect to the direction of motion) of the DoT is controlled by the signal provided by protein A. That is, the maintenance of *A-*mRNA, whose concentration is constant inside the DoT, is switched off (cells forming the DoT differentiate) when the concentration of protein *A* achieves the threshold value *T_A_*. In terms of the model (1–2) and its stationary solution (3–4) this gives:
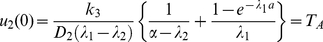
(8)and, therefore, the size of the DoT, *a*, is not a preset parameter but a function of other model parameters, including *T_A_*:
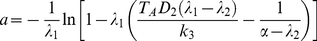
(9)


Furthermore, the threshold value, *T_A_*, is generally achieved in two points (on the either side of the maximum whose location is given by (6)). As the concentration of *u_2_* should not get above *T_A_* in the DoT the differentiation should take place before the maximum is achieved, i.e. condition (7) is to be held. Combining equation (9) with the inequality (7) we define the condition when the stationary solution to the stated problem exists:
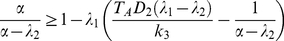
(10)


An important case to consider is when the concentration of the protein A is low and doesn't reach the threshold value, *T_A_*, anywhere in the medium. In the simulations we presume for this case that the size of the DoT is increasing over time (due to proliferation of cells) and the coordinate of the DoT's left border is gradually decreasing (the degree of “graduality” represents the proliferation rate). Simulations show that the size of the DoT is fixed and stable under the condition given by the equation (10). Furthermore, simulations show that if this condition does not hold the size of the DoT oscillates over time (see Figure 3 and Movies S2 and S3).

#### Four-variable models

Modeled protein A down-regulates its own transcription while experimental results shown in Figure 4 indicate that *FGF8* is not involved in the control of its own transcription. Thus protein A does not correspond to *FGF8* and we need to analyze possible relationships between these two morphogens. We have examined two possibilities:

1. Transcription of *FGF8* is proportional to the concentration of protein A (see Figure 5 A, B). This is expressed in the following equation for the concentration (*u_3_*) of *FGF8* mRNA:
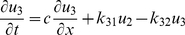
(11)
2. Transcription of *FGF8* mRNA and *A-*mRNA take place in the (nearly) same group of cells: they have been switched on independently from each other but both switched off by the signal provided by protein A. In this scenario the concentration of *FGF8* mRNA is calculated the same way as the concentration of *A-*mRNA in the basic model (see above, equation 1).

In both cases the concentration of *FGF8* protein (*u_4_*) is given by the equation:
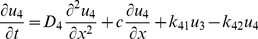
(12)i.e. similarly to the concentration of protein A, *u_2_*, (see equation 2) it is a diffusible agent and its production is proportional to the level of its corresponding gene (*FGF8* mRNA, equation 11) and decays proportional to its own concentration.

#### Modeling chemotaxis

In this version of the model, parameter *c*, defining the DoT migration speed, is calculated with the assumption that the migration is taking place due to chemotaxis, i.e. the speed is proportional to the gradient of the chemotactic agent [42], [43]. We have assumed that protein A acts as a chemo-repellent on cells forming the DoT and the speed of migration is defined either by its gradient in some specific point, say on the front (right-side, *x = a*) of the DoT:
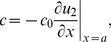
(13)(see Movie S6) or by the average gradient over the DoT,
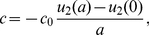
(14)(see Movie S7).

The analysis of conditions when the DoT can migrate due to self-repulsion is relatively simple when we consider the chemotactic movement of a DoT of fixed size, *a*, i.e. consider solution (3–4) remove condition (8) and add condition (14) which gives:
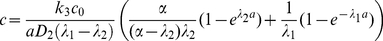
(15)Where the right hand side is also function of *c* (*λ_1_*, *λ_2_* and *α* are functions of c, see the definitions given by (5)). When *c = 0* the right hand side of (15) is zero, i.e. one stationary solution (with *c = 0*) exists for all sets of parameters. One can show that the right hand side of (15) is positive and tends to zero when c tends to infinity. Traveling solutions correspond to the points where *c≠0* and plots of functions
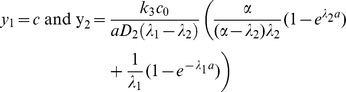
intersect. At least one such point exists, if the derivative of the function *y_2_* (the derivative of the RHS of (15)) is more than 1 at *c = 0*. This condition can be expressed by the formula:
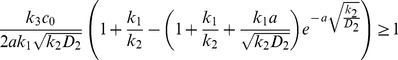
(16)Therefore for sets of model parameters satisfying (16) (see Figure 8A) we can expect the existence of a moving DoT, moving with constant speed. Whether more than one such solution exists and whether such solutions exist when condition (16) is violated should be rigorously analyzed in a more detailed study. We have plotted the function given by the RHS of (15) versus variable *c* for various sets of model parameters. It looks that this function always has only one maximum. Therefore we expect that inequality (16) gives the condition for the Pitch-Fork bifurcation, i.e. we have only one solution (corresponding to c = 0) when model parameters do not satisfy (16) and two extra solutions appear (corresponding to the DoTs moving in opposite directions) when (16) is satisfied. But indeed this conclusion should be verified by proper analysis.

#### Details of simulations and verification of parameters

For simulations we used the explicit Euler's method with central differencing scheme for diffusion and alternating up- and down-wind schemes for advection. Typical initial conditions: all concentrations are equal to zero with the only exception: *u_1_ = 1* in the DoT which has a predefined location and size. Default values of the parameters: diffusion coefficients *D_2_ = D_4_ = 0.5*; kinetics rates *k_1_ = k_31_ = k_41_ = 0.0003*, *k_2_ = 0.00025*, *k_3_ = 2k_2_*, *k_32_ = k_42_ = 2k_31_*; speed *c = 0.015*, chemotaxis *c_0_ = 2*.

Default values for the time and space steps *ht = 1* and *hx = 1* for which we found the simulations to be fairly accurate: two-fold reduction of space step together with four-fold reduction of time step (*ht = 0.25* and *hx = 0.5*) was altering measured quantities (such as maximums in concentration profiles, the DoT size when differentiation is on, and the DoT speed when it is drove by chemotaxis) by less than 3%. Also the simulations were performed in sufficiently large domain to reduce the influence of medium boundaries (doubling the size of the medium has changed measured quantities by less than 1%).

To scale the model parameters we estimate the DoT size to be *1 mm* and its speed to be *0.1 mm/hour*. Comparing this with the simulations shown on Figure 3 where *c = 0.015* (space units over time units) and the DoT size is *40* (space units) we conclude that the space unit corresponds to about 20 µm and the time unit - to 10 seconds. This means that D = 0.5 corresponds to *2·10^−7^ sm^2^/sec* and kinetics coefficient *k = *0.0003 – to *3·10^−3^ sec*.

A few words about justification of the parameter values used in our simulations. Firstly, the analysis of the model represented by equations (1) and (2) with extra conditions (8) and/or (14) indicates that qualitatively the solution is the same for any set of parameters represented by positive numbers. Furthermore, we can take three arbitrary numbers to represent the values of three parameters appropriate for scaling dimensions associated with time, distance and concentration. In our case we decided that the concentration of *A*-mRNA inside the DoT is 1, the DoT is represented by about 40 grid points (or its size is 40 space units if the grid size is 1) and the speed of the DoT is something between 0.1 and 0.01. The choices for the DoT size and speed are dictated by the accuracy issue. We have checked that 40 grids for the DoT gave considerably more accurate solutions than say 10 grids and, on the other hand, approximately the same accuracy as 100 grids. Similarly, if we assume that the time step is 1 then speed c should be less than 0.1 (say 1/40) to provide enough accuracy in numerical calculation of concentration profiles. Diffusion *D = 0.5* is convenient when it comes to the numerical scheme (the highest possible value when the explicit Euler scheme with time and space steps *ht = hx = 1* is still stable) and still in a range of diffusion coefficients known for proteins. Kinetic rate *k_1_* has been chosen in a way that the space scale for the mRNA degradation is comparable with the size of the DoT: this is done to fit with the observations concerning the sizes of FGF8 mRNA transcription and expression (Figures 1 and 4). Other kinetic constants have been chosen to be of the same order as *k_1_*. And finally, concerning *k_2_* and *k_3_*: the ratio of these two constants is only important for the choice of the threshold value *T_A_*: the ratio 2 has been chosen only to bring concentrations of *A*-mRNA and protein A to the same scale (Figures 2, 3, 5).

### Glazier-Graner-Hogeweg Model

This is a computational individual-based model originally developed by Graner and Glazier [26], [27]. In this model we consider the DoT as a group (25 by default) of cells, each, in turn, is represented by a number of grid points (50 grid points per cell in our simulations) on a regular (square-shaped 2D in our case) lattice (see also Methods Section in [8]). Movement of a cell (or change in its shape) means that the cell looses or gains some grid points on the lattice. In terms of the underlying tissue this implies that the grid points are associated with different cells at different times. To calculate whether a particular grid point will be associated with a different cell at next time step a variation principle is used to minimize a quantity representing “the energy” of the system.

Contrary to the original implementation of the GGHM which was based on Monte Carlo algorithm involving the random choice of the pixel followed by the random choice of its neighbor and following calculations of probability of change [26] we have implemented a synchronous model: at each time step we calculate the probability to change the state for all grid points. For each grid point, we randomly select a neighbor (one out of the eight nearest) and calculate how the energy of the system will change after changing the state of the grid point to that of its neighbor. If this change results in an energy decrease we allow the change to occur; if the energy is increased we calculate the probability of that change, *p*, using the Boltzmann function: *p = exp(−ΔE/T)* where the parameter *T* can be referred to as the “temperature” of the system.

The energy is defined in a way that its change accounts for the work done by different forces acting upon moving or deforming cells. The definition of energy used in our implementation of the model takes into account three forces, the adhesive forces between cells, the force associated with the incompressibility of cells (pressure) and forces developed by chemotacticaly moving cells:
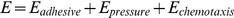
(17)


The following definitions of the terms on the right hand side of equation (17) are commonly used in various modifications of GGHM [8], [28]:

1. An adhesive energy associated with cell-to-cell contacts is defined by the adhesion matrix *J_k,l_* (*J_k,l_* = *J_l,k_*) which refers to an interface between neighboring grid points which belong to different cells (numbers *k* and *l* represent cell types of these cells). The energy, *J_k,l_*, characterizes the strength of a particular cell's adhesive contacts (stronger contacts correspond to smaller energies). To consider adhesive contacts between cells and the surroundings we treat the letter as a special cell of its own type. In our simulations we, as a rule, consider 3 cell types: the surrounding was considered as a cell of its own type – cell type *1*; cell type *2* – cells which form the DoT; cell type *3* – cells which form the DoT trail or the differentiated daughter cells. The default adhesion matrix for adhesive bonds between each pair of different cell types is:
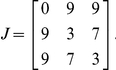

2. To control the size of a cell (say *k^th^* cell), *A_k_(t)*, a target area (in case of our 2D cells number of grid points forming the cell can be considered as its area), *T_k_*, is introduced. The *k^th^* cell is given an energy *E_vol,k_ = α(A_k_(t)−T_k_)^2^*, where *α* is a positive constant (*α = 0.6* in all our simulations). Constant *α* represents the cell's resistance to compression and we can call it the “incompressibility” coefficient (it had different names such as “Lagrange multiplier specifying the strength of the area constraint” in [26], or “volume elasticity” in [44]) referring to another approach when the motion of cells in a tissue is seen as a flow in incompressible viscous liquid described by Navier-Stokes equation [45], [46] and where this term would correspond to the gradient of pressure. The “incompressibility” energy reaches its minimum (zero) when the cell's actual and target areas are equal. To take into account the growth of cells the target area, *T_k_*, is considered to be an increasing function of time. To model cell proliferation we split big cells (which contain 100 or more grid points) into two small cells. The split is performed along a straight line (having a random orientation) crossing the cell's centre of mass.
3. To implement the chemotactic effect of the agent, whose concentration is denoted as ‘*u*’, to a moving (or deforming) cell we introduce the change in chemotactic energy Δ*E_ch,k_ = β_k_ (* ***x grad*** *(u))*, where *β_k_* is a constant describing the chemotactic response of cells of type *k* to the chemotactic agent *u* and ***x*** is a vector representing the local displacement of the given cell's boundary. This energy change refers to the work done by chemotactic force Δ*E_ch,k_ =* ***F*** *_ch,k_·* ***x*** and therefore corresponds to the chemotactic force, ***F*** *_ch,k_* = *β_k_* ***grad*** *(u)*, exerted by chemotactically responding cell. The identical definition of the chemotactic force was introduced and used earlier in the hydrodynamic model of Dictyostelium development [47]. The most common implementation of chemotaxis in our model: *β_k_ = 0* if *k≠2* and *β_k_ = β* (*β≠0*) for *k = 2* or for “red” cells forming the DoT. This means that there is an energy gain or loss in the system related to the relocation of the red cell's boundary which depends on the local gradient of chemotactic agent. We note that a positive value for the parameter *β* corresponds to the process of chemorepulsion while a negative value for *β* - to chemoattraction.

Detailed description of the GGHM model and its modifications and applications to various problems in developmental biology are given in [28]. One of the greatest advantages of GGHM is that it allows modeling the dynamics of biological tissue while being focused on behavior of individual cells. The simplicity of the model allows modeling of tissue which contains up to 10^5^ cells on a single PC. Parallel implementation of the software [44] allows an increase in this number up to 10^7–^10^8^ which is close to the actual number of cells in many real tissues. Furthermore, the GGHM allows relatively simple modifications to address various problems associated with mechanics and deformations of cells. For example the GGHM allows consideration of cells of different shapes. Cells in the version of the GGHM which we use here are predominantly round-shaped. To model, for example, elongated cells the GGHM can be extended by the introduction of the anisotropy in adhesive properties of cells [48] or by the introduction of cellular subunits which compose cells of desired shape and stiffness [49]. The GGHM has also been extended to address three-dimensional problems [50] and its simulation code is available publically (the CompuCell3D package at http://www.compucell3d.org).

#### Details of simulations

We have implemented a synchronous model: at each time step we calculate the probability to change the state for all grid points. For each grid point, we randomly select a neighbor (one out of the eight nearest) and calculate how the energy of the system will change after changing the state of the grid point to that of its neighbor. If this change results in an energy decrease we allow the change to occur; if the energy is increased we calculate the probability of that change, *p*, using the Boltzmann function: *p = exp(−ΔE/T)* where the parameter *T* can be referred to as the “temperature” of the system (we set *T = 6* in all our simulations).

All simulations start with a group of 25 cells (forming an artificial square-shaped tissue) representing the DoT. In the simulations where we do not consider cell proliferation, we assume that all cells have a constant target volume (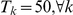) which does not depend on the cell age. In the simulations where we take into account cell growth and proliferation, we assume that the initial target volumes of cells are randomly distributed among the cells in the range (30–70) and then the target volume of each cell is increased by one unit every 10 time steps with probability 1/3. When the actual volume of a cell reaches 100, the cell divides along a line crossing through the cell's centre of mass with a random direction of the cleavage plane. After division the target volumes of both daughter cells are reset to 50 and they start to increase again over time. This implies that the average time required for a cell to double in size and proliferate is equal to 1500 time steps. One time step scales as 70 seconds (as it derived in the next section) and therefore the effective proliferation rate in the model is one division per 30 hours. In experimental conditions the proliferation rate is much higher (one division per 6 hours) but on the other hand,, in experimental conditions, many cells leave the stem zone (and epiblast) and transform into mesenchyme cells. Since in our model we don't consider formation of mesenchyme cells (this would require three-dimensional version of the model) we have to reduce the proliferation rate of cells in the epiblast (to compensate the mesenchyme formation). Furthermore we did not consider the proliferation of differentiated (green) daughter cells as this would not influence the phenomena which we are interested in but add unnecessary details into simulations and graphical outputs used in the figures.

#### Verification of parameter values in GGHM

Parameters used in the GGHM can be split into two sets. One set is used for the definition of energy in the system and is associated with adhesiveness (entries *J_k,l_* in the adhesion matrix), incompressibility (parameter *α* defining incompressibility) and chemotactic responses of cells (parameter *β* defining chemotactic response) as well as temperature *T* in Boltzmann function. The second set of parameters is used to define the dynamics of morphogen concentrations (kinetics and diffusion of morphogens). The First set of parameters forms a core of the GGHM and verification of the parameter values used for this set can be found in the literature ([27], [28] including more references in [28]). Here we can briefly note that the most important point concerning the entries *J_k,l_* in the adhesion matrix is their ratios: *J_k,1_* = *J_1,k_*>2 *J_k,k_* for cells to stay together and form a tissue. Also *J_k,l_* = *J_l,k_*>2 *J_k,k_* and *J_k,l_* = *J_l,k_*>2 *J_l,l_* for cells of types *k* and *l* to sort out or to stay sorted out. The values of the entries *J_k,l_* are scaled with the values of parameters *α* and *β* in order to scale all three considered forces (associated with adhesion, pressure and chemotaxis) relative to each other. The value of parameter *T* defining the rate of the evolution in the system is also scaled with the values of *J_k,1_*, *α* and *β*. The ratio *α/T* defines the amplitude of the cell shape fluctuations (or cell membrane fluctuations). These fluctuations freeze at high values of *α* as well as at low values of the Boltzmann temperature *T*. If we will keep all parameters of the model constant and vary only the temperature we will see that the rate of dynamics in the model will be low at low temperatures, then the processes accelerate with the increase of the temperature and eventually they slow down again when the temperature becomes too high. We have measured the speed of migrating group of cells as a function of the Boltzmann temperature (keeping all other model parameters at their “default” values) and found that the highest speed is observed at *T = 6* (see Figure S1). It was noted in [51] that the Boltzmann temperature, *T*, defines the intrinsic cell motility in GGHM. Therefore *T = 6* (which we have chosen for our simulations) corresponds to the highest possible intrinsic cell motility for the given set of other model parameter values.

It was shown on many occasions that the GGHM is robust: small variations in the values of model parameters do not alter qualitatively the outcome of simulations. Besides, it was shown that the GGHM parameters can be rescaled so that the outcome of simulations is absolutely the same. For example, the simulation of the primitive streak progression was performed in [8] in tissues containing 625 and 15000 cells without any notable difference in the outcome.

The concentration fields of morphogens were calculated in a way similar to that for the 1D model. The level of *A-*mRNA was set equal to 1 in all (red) cells forming the DoT and was decaying in differentiated (green) cells according to the equation: 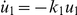 similar to what was in the 1D (compare with the equation 1 where *c = 0*). The equation for protein *A* includes diffusion, production and decay and is given by the equation 2 (see above) where *c = 0*. There are no advection terms in the 2D model as the events are considered in the laboratory frame of reference. As the GGHM is considerably slower (as compared with our 1D model) we have increased the speed of computations by ensuring slightly faster processes (faster moving DoT and faster kinetics for chemicals). For *k_1_ = 0.001*, *k_2_ = 0.003* and *β = 4.5* (see Figure 6) the speed of the DoT is roughly 60 space steps per 1000 time steps (should correspond to 0.1 mm/hour) and the DoT size is roughly 32 grid points (should correspond to 1 mm). This means that 1 space unit roughly corresponds to 30 µm, 1 time step to 70 seconds causing for dimensional diffusion and kinetic coefficients to be slightly (2 to 3 times) less than for the set of parameters used in the 1D model.

### Experimental Methods

Stage Hamburger and Hamilton (HH) 9–10 chick embryos were obtained from fertilized eggs (Granja Santa Isabel, Cordoba, Spain) and dissected in L15 culture medium (Invitrogen).

Embryos were cultured in 4 well dishes on top of collagen beds and with 0.2 ml of culture medium (Optimem (Invitrogen), fetal calf serum, glutamax and gentamicine) containing 0.1% DMSO (control) or PD173074 (10 µM in 0.1% DMSO, Sigma). Caudal explants (including 3 embryonic layers) were cultured in collagen as described in [52] in the presence of BSA (control) or hFGF4 (330 ng/ml, Sigma). For splitting the caudal domain into two, embryos were prepared following the EC culture method [53], a cut was performed caudal to the node with a microsurgical knife and embryos where cultured for another 20 h.

Embryos and explants were fixed in 4% PFA and processed for in situ hybridization with probes to detect either nascent [15] or total *FGF8* following standard methods.

## Supporting Information

Figure S1 **The effect of Boltzmann temperature, T, on the speed of the migrating DoT.** The plot is produced under the set of assumptions used for the simulation shown in Figure 6 and Movie S4. At T = 0 the DoT does not migrate (cell shapes are frozen). A temperature increase induces the DoT migration (allows cell shape fluctuations) and the DoT's speed increases until reaching a maximum when T = 6. After this (for T>6) the speed gradually decreases with the increase of the temperature, indicating that the further amplification of the cell shape fluctuations reduces cell's motility. Therefore the Boltzmann temperature can be seen as a parameter defining intrinsic motility of cells with a maximum at T = 6 (when other model parameters are fixed at values used in Figure 6).
(TIF)

Table S1 **Summary of simulation results for both models and all considered sets of model assumptions.** Using the continuous one-dimensional and individual-based two-dimensional models we have considered migration of the domain of transcription (DoT) under a few distinct sets of assumptions concerning proliferation, differentiation and movement of cells forming the DoT. The summary of mechanisms with the references to the figures and supplementary movies demonstrating simulation outcomes has been provided.
(TIF)

Movie S1 Formation of stationary concentration profiles in the basic model (transition from the initial conditions to the stationary solution (3–4) to the equations (1–2) in Materials and Methods Section).
(MPEG)

Movie S2 Formation of stationary concentration profiles of gen *A-*RNA and protein A in the model with the DoT size regulation (see Materials and Methods Section). The regulation of the DoT size is implemented the following way: if the concentration of the protein A does not achieve the threshold level *T_A_* all over the medium the DoT size increases with a constant rate (the DoT's left border shifts to the left with a constant rate), otherwise the DoT's left border is at the right-most point where the level of the protein A is equal to the value of *T_A_*. To obtain a smooth dynamics the size of the DoT was fixed (like in the basic model), i.e. the regulation of the DoT size was switched off for the first 3000 time steps.
(MPEG)

Movie S3 Oscillations in concentration profiles of *A*-RNA and protein A in the model with the DoT size regulation (see Materials and Methods Section). The model used here is the same as that used to produce Movie S2 (and Figure 3). Model parameters are the same as for Figure 2 except for the value of parameter *k_1_*: *k_1_ = 0.008*.
(MPEG)

Movie S4 Concentration profiles of *A*-mRNA and protein A in the model with the DoT speed defined by the chemotaxis (see Materials and Methods Section). Chemotaxis is defined by the gradient of protein A on the front (right border) of the DoT. All other features of the model, except for *k_1_ = 0.045*, are the same as in Movie S2 (and Figure 3A).
(MPEG)

Movie S5 Concentration profiles of *A*-mRNA and protein A in the model with the DoT speed defined by the chemotaxis (see Materials and Methods Section). Chemotaxis is defined by the average gradient of protein A over the DoT. To initiate the moving DoT we have used the following procedure: the size and the speed of the DoT were fixed (exactly as in Movie S2) for the first 3000 time steps and only after that the differentiation and chemotaxis were switched on.
(MPEG)

Movie S6 The DoT migration in the GGHM. The DoT is represented by group of red cells moving to the right side. Red cells proliferate and differentiate, i.e. transform into the green cells which do not move and where *A*-mRNA decays. Cell differentiation is regulated by the level of protein A (as in Figure 3, and Movies S2 and S3).
(MPEG)

Movie S7 The DoT migration in the three-cell-types version of the GGHM. The DoT is represented by group of red cells moving right-wise. Red cells proliferate and differentiate, i.e. transform into the green cells which do not move and where *A*-mRNA decays. Cell differentiation is regulated by the level of protein A.
(MPEG)

Movie S8 When the kinetics of the protein is too slow the “self-repelled” DoT meanders. The DOT is “forced” to move right-wise (see about the DoT with preset motion in the Results Section) for 2000 time steps to provide with the initial conditions. After this the preset motion is switched off and the DoT is repelled by the transcribed protein.
(MPEG)

Movie S9 When the kinetics of the protein is neither slow nor fast the “self-repelled” DoT moves along a line (orientation of the line is defined by the initial conditions). The DoT is “forced” to move right-wise (see about the DoT with preset motion in the Results Section) for 2000 time steps to provide with the initial conditions. After this the preset motion is switched off and the DoT is repelled by transcribed protein.
(MPEG)

Movie S10 When the kinetics of the protein is too fast the “self-repelled” DoT moves and deforms. The DoT is “forced” to move right-wise (see about the DoT with preset motion in the Results Section) for 2000 time steps to provide with the initial conditions. After this the preset motion is switched off and the DoT is repelled by the transcribed protein.
(MPEG)

Movie S11 Chemotactic migration of the DoT when its constituent cells grow, proliferate and differentiate.
(MPEG)

Movie S12 Simulation of the cutting experiment (see Figure 10).
(MPEG)
