# Supplementary figures and images for: Coordination of Cell Differentiation and Migration in Mathematical Models of Caudal Embryonic Axis Extension
Source: PLoS One. 2011 Jul 28;6(7):e22700. doi: 10.1371/journal.pone.0022700 (PMC3145656; doi:10.1371/journal.pone.0022700)

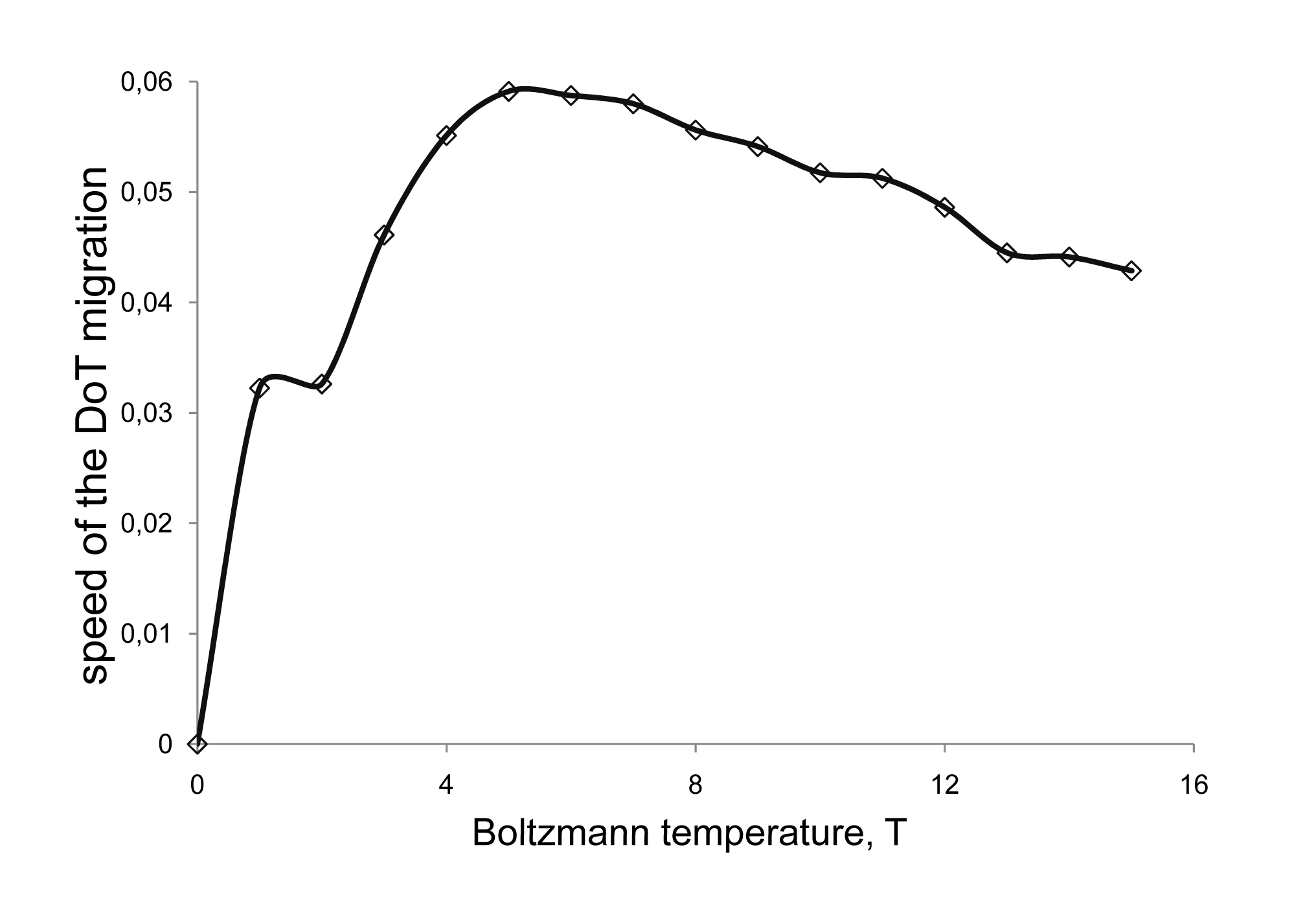

Supplement: Figure S1 — The effect of Boltzmann temperature, T, on the speed of the migrating DoT. The plot is produced under the set of assumptions used for the simulation shown in Figure 6 and Movie S4. At T = 0 the DoT does not migrate (cell shapes are frozen). A temperature increase induces the DoT migration (allows cell shape fluctuations) and the DoT's speed increases until reaching a maximum when T = 6. After this (for T>6) the speed gradually decreases with the increase of the temperature, indicating that the further amplification of the cell shape fluctuations reduces cell's motility. Therefore the Boltzmann temperature can be seen as a parameter defining intrinsic motility of cells with a maximum at T = 6 (when other model parameters are fixed at values used in Figure 6). (TIF) [file pone.0022700.s001.tif]

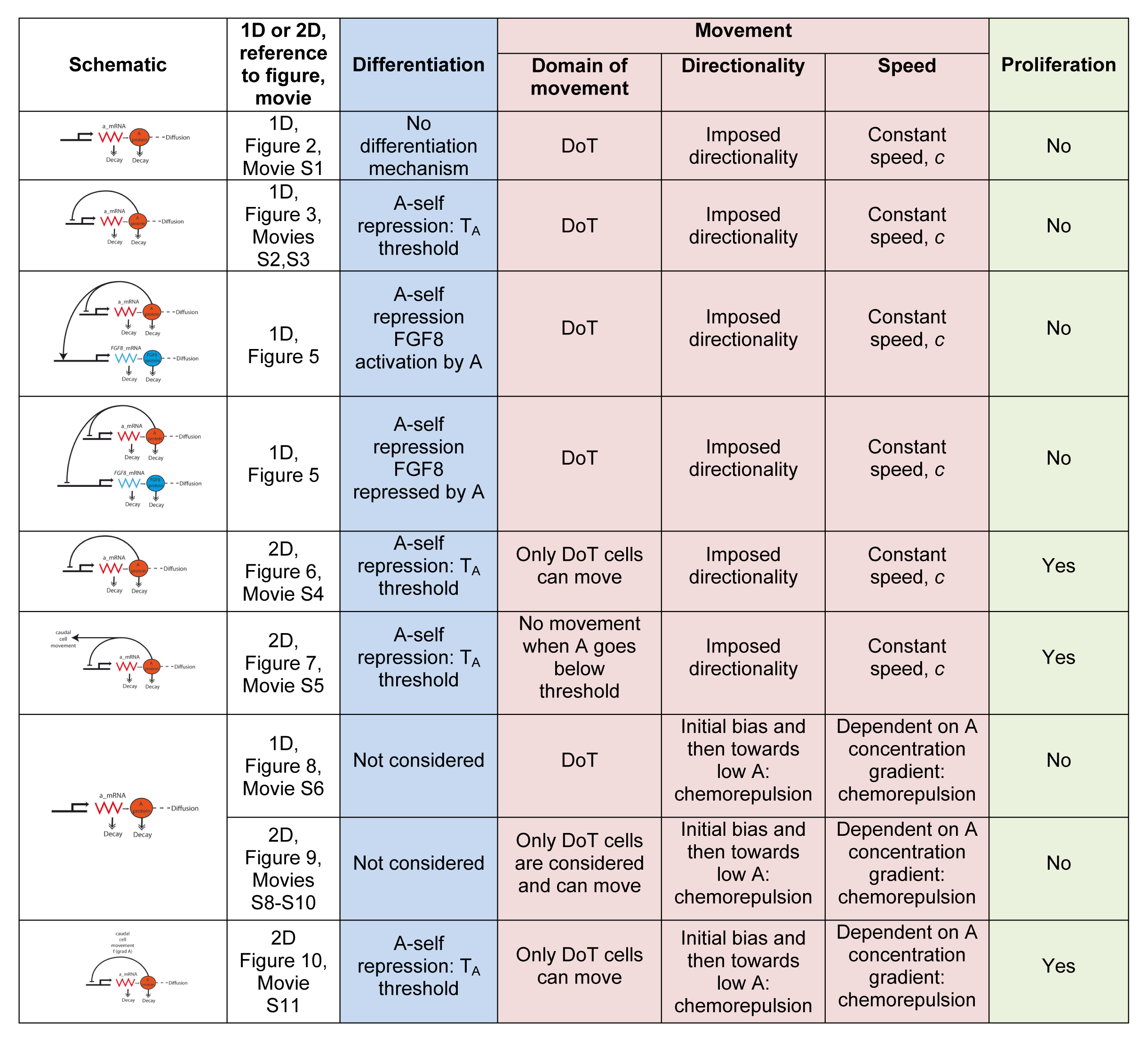

Supplement: Table S1 — Summary of simulation results for both models and all considered sets of model assumptions. Using the continuous one-dimensional and individual-based two-dimensional models we have considered migration of the domain of transcription (DoT) under a few distinct sets of assumptions concerning proliferation, differentiation and movement of cells forming the DoT. The summary of mechanisms with the references to the figures and supplementary movies demonstrating simulation outcomes has been provided. (TIF) [file pone.0022700.s002.tif]
